# Nanoparticle-Based Lateral Flow Biosensors Integrated With Loop-Mediated Isothermal Amplification for the Rapid and Visual Diagnosis of Hepatitis B Virus in Clinical Application

**DOI:** 10.3389/fbioe.2021.731415

**Published:** 2021-09-14

**Authors:** Xu Chen, Shoshi Wang, Yan Tan, Junfei Huang, Xingui Yang, Shijun Li

**Affiliations:** ^1^ The Second Clinical College, Guizhou University of Traditional Chinese Medicine, Guiyang, China; ^2^ Central Laboratory of the Second Affiliated Hospital, Guizhou University of Traditional Chinese Medicine, Guiyang, China; ^3^ Guizhou Provincial Center for Clinical Laboratory, Guiyang, China; ^4^ Laboratory of Bacterial Infectious Disease of Experimental Centre, Guizhou Provincial Centre for Disease Control and Prevention, Guiyang, China

**Keywords:** hepatitis B virus, loop-mediated isothermal amplification, lateral flow biosensor, rapid of detection, limit of detection

## Abstract

Hepatitis B virus (HBV) infection remains one of the major public health issues worldwide. Developing a rapid, sensitive, specific, easy-to-operate, and cost-saving approach for the diagnosis of HBV is essential for its therapy and prevention. Here, we first devised a novel approach, termed “loop-mediated isothermal amplification integrated with a nanoparticle-based lateral flow biosensor (LAMP-LFB),” for the detection of HBV in clinical application. The results indicated that a set of LAMP primers based on the *S* gene were valid for the establishment of HBV-LAMP-LFB. The optimal HBV-LAMP can be carried out at a constant temperature of 65°C for 40 min. The whole detection process, including HBV genomic DNA preparation (∼10 min), LAMP (40 min), and LFB reading (within 2 min), can be accomplished within 60 min. The limit of detection of the HBV-LAMP-LFB assay was 7.5 IU per test. The specificity of this assay was one hundred percent, and there was no cross-reactivity with other pathogens. Hence, these results indicated that the HBV-LAMP-LFB assay established in the current study is a sensitive, rapid, specific, visual, simple, and cost-saving method for the screening of HBV agents. More importantly, the HBV-LAMP-LFB has remarkable potential to develop a point-of-care testing in clinical application, especially in resource-scarce regions.

## Introduction

Hepatitis B virus (HBV) is a life-threatening pathogen that can cause acute and chronic infection and puts people at high risk of death from cirrhosis and liver cancer (Bollinger et al., 2020; Liaw, 2019). Generally, HBV spreads through blood or blood-related products from HBV-infected patients. Most people do not experience any symptoms when newly infected with HBV. When the disease progresses, some people show jaundice, extreme fatigue, nausea, acute liver failure, hepatitis, liver cirrhosis, and hepatocellular carcinoma (Blackard and Sherman, 2018; Revill et al., 2019). It is hard to detect HBV-infected cases, owing to the absence of hepatitis B surface antigen and a low amount of HBV-DNA in blood at the early stage of HBV infection (Liang, 2009; Tong and Revill, 2016). The problem mainly compounded by the lack of advanced diagnostic methods and medical services for donor screening or routine testing of patients. Hence, developing a rapid, sensitive, specific, easy-to-use assay is critical for addressing the burden of HBV infection.

Traditionally, enzyme-linked immunosorbent assay (ELISA) and real-time quantitative polymerase chain reaction (qPCR) have been widely used to probe HBV based on HBsAg and HBV-DNA (Mallika, 2015; Villar et al., 2015). However, the sensitivity of the ELISA was remarkably low, especially in the early stage of HBV infection (Sara, 2011). In recent decades, nucleic acid amplification technologies (NAATs), including polymerase chain reaction (PCR) and real-time quantitative PCR were considered as good alternative methods for the diagnosis of HVB infection owing to being more sensitive than ELISA (Jain et al., 2018; Coffin et al., 2019). Nevertheless, these methods need special laboratory layout and expensive instruments that may not be readily available in many resource-shortage settings. Moreover, it is time-consuming (approximately 130 min). Herein, developing a cost-effective, rapid, simple, sensitive, and specific assay for identification of HBV is necessary for the diagnosis of blood donors and HBV-infected patients.

To address the shortcomings of these approaches, loop-mediated isothermal amplification (LAMP), a rapid and simple NAAT, has been devised and gradually applied to the diagnosis of many pathogens, such as human papillomavirus, influenza virus, and severe acute respiratory syndrome coronavirus 2 (Zhu et al., 2020; Landaverde et al., 2020; Le Thi et al., 2020), which can robust amplify the target sequence at a fixed temperature (58–69°C) for 30–60 min. The primer set includes a pair of outer primers (F3 and B3), loop primers (LF and LB), and inner primers (FIP and BIP) (Seki et al., 2018). Then, analysis of the LAMP reactions has become another major concern. Conventionally, agarose gel electrophoresis, fluorescent agents, real-time turbidimetry, and colorimetric indicators (MG reagent) have been widely used to read out LAMP products (Romero and Cook, 2018; Kubota and Jenkins, 2015; Notomi et al., 2000). However, these methods require special facilities and reagents. To overcome these drawbacks, the gold nanoparticle-based lateral flow biosensor (LFB) with easy accessibility, good robustness, visualization, low limits of detection, and specificity features has been reported for the detection of nucleic acid and protein molecules in recent years (Quesada-Gonzá lez and Merkoçi, 2015; Huang et al., 2021).

**FIGURE 1:**
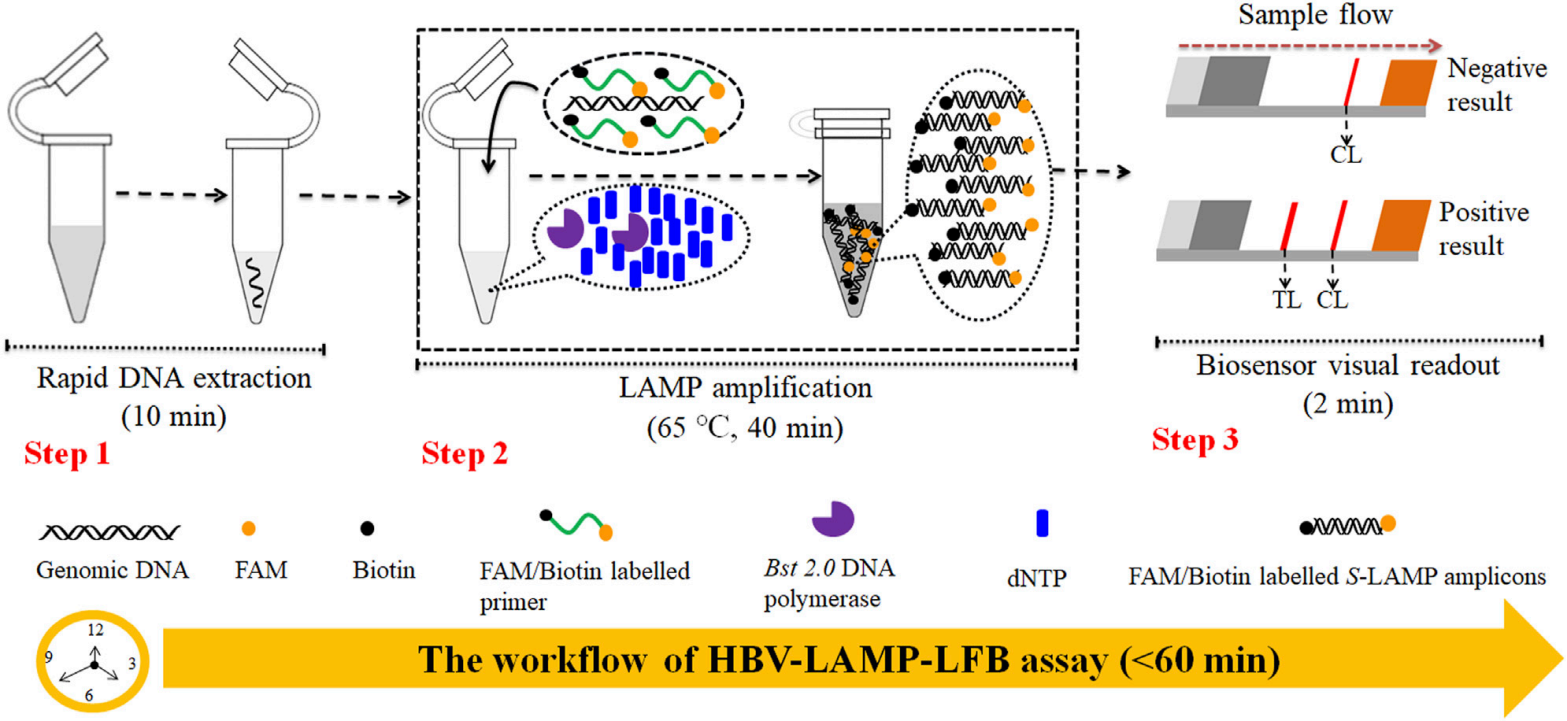
Outline of the HVB-LAMP-LFB workflow. The HVB-LAMP-LFB assay system contains three closely linked steps: DNA extraction (step 1), LAMP (step 2), and gold nanoparticle-based LFB readout (step 4). The whole detection process can be completed within 60 min.

In this study, the loop-mediated isothermal amplification integrated with the gold nanoparticle-based lateral flow biosensor (LAMP-LFB) was devised first for rapid, visual, simple, specific, and sensitive identification of HBV by targeting on the *S* gene (Nyan et al., 2014). The principle of the HBV-LAMP-LFB is illustrated in Figure 1, and its feasibility was validated with clinical samples.

## Materials and Methods

### Standard Panels and Clinical Samples Preparation

HBV, hepatitis C virus (HCV), and human immunodeficiency virus (HIV) nucleic acid standard substances were obtained from the Chinese Academy of Metrology (Code No. GBW09150, GBW(E)090118, and GBW(E)090272, respectively). 115 clinical serum specimens were collected from patients with suspected HBV infection at the Second Affiliated Hospital, Guizhou University of Traditional Chinese Medicine (Guizhou, China), during June 2019 to May 2020 and stored at −80°C until use; other non-HBV pathogens used in the current study are shown in Table 1. The genomic DNA templates were extracted using nucleic acid-releasing agents (Sansure Biotech, Changsha, China) in accordance with the manufacturer’s instructions and stored at −20°C before use. The concentrations were measured using NanoDrop ND-2000 (Beijing, China) at A260/280.

**TABLE 1:** Pathogens used in this study.

No.	Pathogen	Pathogen no. (source of pathogens)^a^	No. of strains	LAMP-LFB result^b^
1	HBV (standard substance)	The Chinese Academy of Metrology	1	P
2	HBV(clinical samples)	2nd GZUTCM	13	P
3	HCV (standard substance)	The Chinese Academy of Metrology	1	N
4	HIV (standard substance)	The Chinese Academy of Metrology	1	N
5	H1N1 (nucleic acid samples)	GZCDC	1	N
6	Influenza B	GZCDC	1	N
7	Respiratory syncytial virus type A	GZCDC	1	N
5	Human rhinovirus	GZCDC	1	N
7	Adenoviruses	GZCDC	1	N
8	*Mycobacterium tuberculosis*	GZCDC	1	N
9	*Pseudomonas aeruginosa*	2nd GZUTCM	1	N
10	*Mycoplasma pneumoniae*	2nd GZUTCM	1	N
11	*Streptococcus pneumoniae*	2nd GZUTCM	1	N
12	*Mycoplasma pneumonia* M129/FH	2nd GZUTCM	1	N
13	*Haemophilus influenzae*	ATCC49247	1	N
14	*Streptococcus pyogenes*	2nd GZUTCM	1	N
15	*Staphylococcus aureus*	2nd GZUTCM	1	N
16	*Acinetobacter baumannii*	2nd GZUTCM	1	N
17	*Leptospira interrogans*	GZCDC	1	N
18	*Shigella flexneri*	2nd GZUTCM	1	N
19	*Bacillus cereus*	GZCDC	1	N
20	*Bordetella parapertussis*	GZCDC	1	N
21	*Bordetella pertussis*	GZCDC	1	N
22	Enteropathogenic *Escherichia coli*	GZCDC	1	N
23	*Candida glabrata*	2nd GZUTCM	1	N
24	*Cryptococcus neoformans*	ATCC14053	1	N
25	*Hemophililus parainfluenzae*	GZCDC	1	N

a 2^nd^ GZUTCM, The Second Affiliated Hospital, Guizhou University of Traditional Chinese Medicine; GZCDC, Guizhou Provincial Center for Disease Control and Prevention; ATCC, American Type Culture Collection.

b P, Positive; N, Negative.

## Materials and Instruments

Microbial nucleic acid-releasing agents were obtained from Sansure Biotech (Changsha, China). The colorimetric indicator (malachite green, MG), isothermal amplification kits, and gold nanoparticle-based LFB were obtained from HuiDeXin Bio-technique (Tianjin, China). The dye streptavidin-coated gold nanoparticles (streptavidin-GNPs) were obtained from HuiDeXing Biotech Co., Ltd. (Tianjing, China); the size of GNPs is 34.46 ± 4.34 nm, and the extinction coefficient of GNPs is 6.0 × 10^9^ M^−1^ cm^−1^ at 506 nm. Biotin-BSA (biotinylated bovine serum albumin) and anti-FAM (rabbit anti-fluorescein antibody) were purchased from Abcam Co., Ltd. (Shanghai, China). The real-time turbidimeter (LA-500) was obtained from Eiken Chemical Co., Ltd. (Japan). The HBV real-time quantification PCR diagnosis kit was obtained from DaAn Gene Co., Ltd. (Guangzhou, China).

### Construction of the Gold Nanoparticle-Based Lateral Flow Biosensor

**FIGURE 2:**
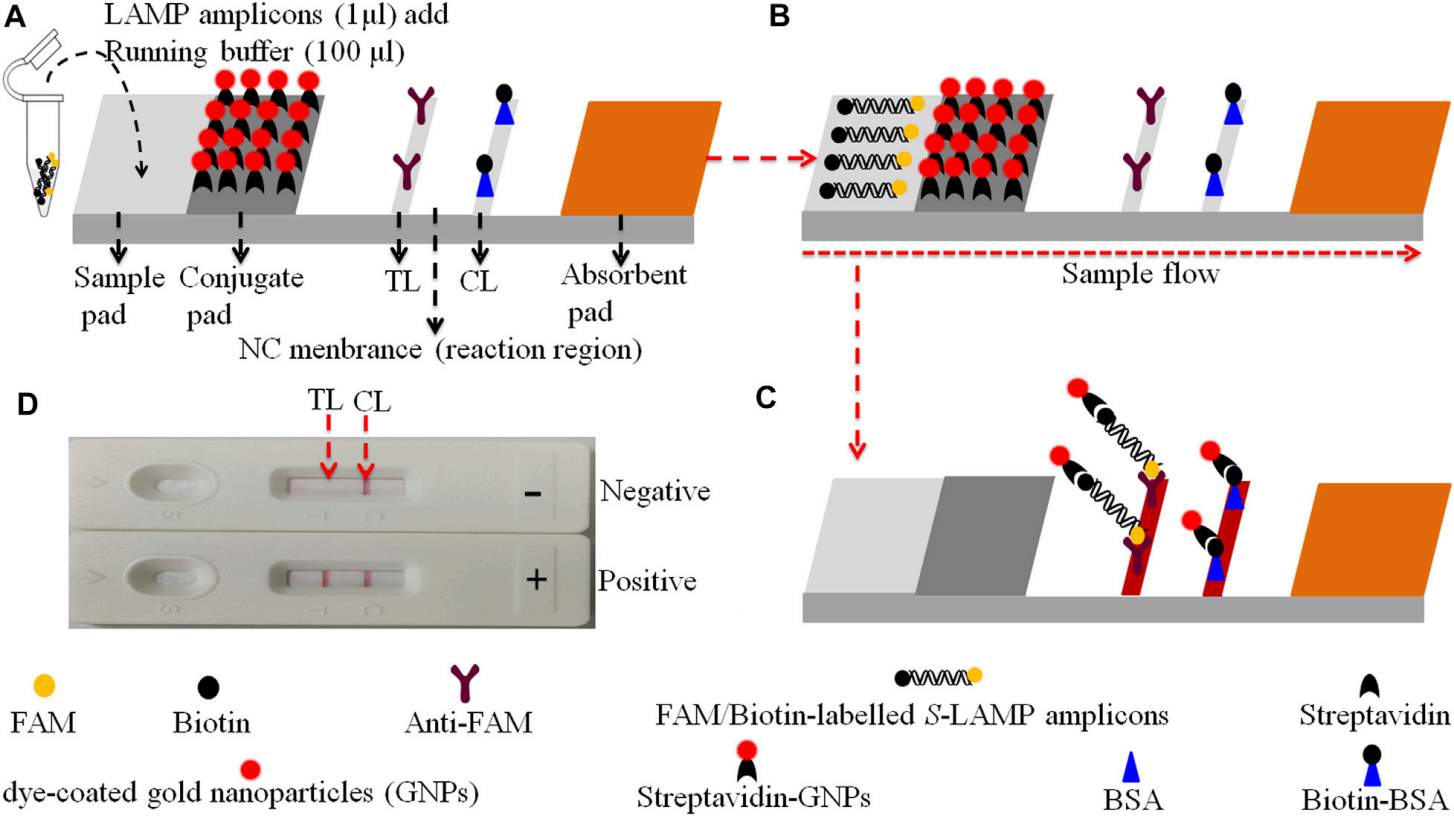
The schematic of the gold nanoparticle-based LFB for the visualization of HVB-LAMP-LFB products. **(A)** The biotin/FAM-labeled HBV-LAMP amplicons (1.0 μL) and the running buffer (100 μL) were added to the sample pad. **(B)** The running buffer containing LAMP products will be moved along the LFB owing to capillary action; meanwhile, the dye streptavidin-coated gold nanoparticles (streptavidin-GNPs) were rehydrated on the conjugate region. **(C)** In the positive sample, the biotin/FAM-labeled HBV-LAMP amplicons were captured by the anti-FAM at the TL and the streptavidin-GNPs were captured by the biotin-BSA at the CL, respectively. However, in negative results, only the streptavidin-GNPs were captured by biotin-BSA at the CL. **(D)** Interpretation of the HBV-LAMP-LFB assay results. For positive results, CL and TL appear on the biosensor, whereas only the CL was observed on the biosensor, indicating negative outcomes.

The gold nanoparticle-based LFB (60 mm × 4 mm) used in the current study is devised and elaborated in Figure 2. In brief, the biosensor consists of four sections, including sample pad, conjugate pad, nitrocellulose (NC) membrane, and absorbent pad (HuiDeXing Biotech Co., Ltd. (Tianjing, China) (Code No. HT100003). The detector reagents (dye streptavidin-coated gold nanoparticles (streptavidin-GNPs), 129 nm, 10 mg·ml^−1^; 100 mM borate, pH 8.5 with 0.05% Tween 20; 0.1% BSA; and 10 mM EDTA) were laminated onto the conjugate pad. Biotin-BSA (4 mg·ml^−1^; Abcam) and rabbit anti-FAM (0.25 mg·mL^−1^; Abcam) were coupled onto the NC membrane for the control line (CL) and test line (TL), respectively. Each band was separated by 5 mm. In the end, the assembled biosensor was preserved in a plastic box and conserved with a silica gel desiccant at room temperature.

### Design and Synthesis of Hepatitis B Virus–Loop-Mediated Isothermal Amplification Primers

**FIGURE 3:**
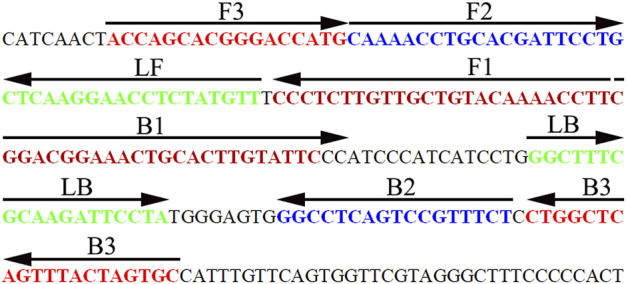
Sequence and location of the HBV-specific *S* gene used to design HBV-LAMP-LFB primers. The nucleotide sequence of the sense strand of the HBV *S* gene is shown in the diagram. Right arrows and left arrows indicate sense and complementary sequences, respectively.

Three pairs of HBV-LAMP special primers based on the *S* gene (GenBank Accession No. AB809557.1) were designed through Primer Explorer V5 and PRIMER PREMIER 5.0 software (Eiken Chemical, Japan). The HBV-LAMP primers were analyzed with the basic local alignment search tool (BLAST) for the primer sets to validate sequence specificity. The position of each primer is shown in Figure 3, and the sequences and modifications are shown in Table 2. All of the oligomers were synthesized and purified by TsingKe Biotech Co., Ltd. (Beijing, China) at an HPLC purification grade.

**TABLE 2:** The primers used in the present study.

Primer name	Sequence and modifications	Length	Gene
F3	5ʹ-ACC​AGC​ACG​GGA​CCA​TG-3ʹ	17 nt	*S*
B3	5ʹ-GCA​CTA​GTA​AAC​TGA​GCC​AG-3ʹ	20 nt	
FIP*	5ʹ-FAM-AAGGTTTTGTACAGCAACAAGAGGG-CAAAACCTGCACGATTCCTG-3ʹ	45 mer	
BIP	5ʹ-CGG​ACG​GAA​ACT​GCA​CTT​GTA​TTC-AGA​AAC​GGA​CTG​AGG​CC-3ʹ	41 nt	
LF*	5ʹ-Biotin-AACATAGAGGTTCCTTGAG-3ʹ	19 nt	
LB	5ʹ-GGC​TTT​CGC​AAG​ATT​CCT​A-3ʹ	19 nt	

FIP*, 5ʹ-labeled with FAM when used in the HBV-LAMP-LFB assay; LF*, 5ʹ-labeled with biotin when used in the HBV-LAMP-LFB assay; FAM, 6-carboxy-fluorescein; nt, nucleotide; mer, monomeric unit.

### The Standard Hepatitis B Virus–Loop-Mediated Isothermal Amplification Reaction

The HBV-LAMP was carried out in a 25 μL reaction system, including 12.5 μL of 2 × reaction buffer (40 mM Tris-HCl (pH 8.8), 40 mM KCl, 16 mM MgSO_4_, 20 mM (NH_4_)_2_SO_4_, 2 M betaine, and 0.2% Tween-20), 1 μL genomic template, 0.4 μM each of outer primer F3 and B3, 1.6 μM each of inner primer FIP* and BIP, 0.8 μM each of loop primer LF* and LB, 1 μL (8U) of *Bst 2.0* DNA polymerase, 1 μL (10U) of AMV reverse transcriptase (for RNA virus only), and 1 µl colorimetric indicator (MG), along with addition of double distilled water to 25 μL. The LAMP was performed at 64°C for 1 h. HCV and HIV were used as negative controls (NCs), and double distilled water (DW) was used as the template in blank control (BC).

### Detection of Hepatitis B Virus–Loop-Mediated Isothermal Amplification Products

Four monitoring techniques, including 2% agarose gel electrophoresis, colorimetric indicator (MG), real-time turbidimeter, and gold nanoparticle-based LFB, were used for the detection and confirmation of the LAMP products. For positive results, the agarose gel presented ladder-like bands and the color changed from colorless to light green in the reaction system. However, there were no bands in gel electrophoresis and it was colorless in NC and BC. For real-time turbidity measurement, turbidity >0.1 is considered as the positive result. With LFB detection, two visible red lines (CL and TL) appeared simultaneously indicating a positive result, and only the CL was observed for a negative outcome.

### Temperature Optimization of the Hepatitis B Virus–Loop-Mediated Isothermal Amplification Assay

Reaction temperature is of vital importance to LAMP. In this study, the reaction temperature was tested in a range from 60 to 67°C with 1°C interval for 60 min. The LAMP products were monitored using real-time turbidity (LA-500). Each temperature was tested independently at least thrice.

### Sensitivity of the Hepatitis B Virus–Loop-Mediated Isothermal Amplification-Lateral Flow Biosensor Assay

To identify the limit of detection (LoD) of the HBV-LAMP-LFB assay, HBV nucleic acid standard substances (Chinese Academy of Metrology) were serially diluted 10-fold from 1.0 × 10^4^ to 1.0 × 10^−1^ IU. The HBV-LAMP operation is as described above, and the LAMP products were analyzed simultaneously with MG and LFB. The sensitivity of HBV-LAMP-LFB was confirmed as the last dilution of each positive test. All examinations were confirmed in triplicate.

### Optimization of Amplification Time for the Hepatitis B Virus–Loop-Mediated Isothermal Amplification–Lateral Flow Biosensor Assay

In order to optimize the reaction time for preamplification of the assay, four reaction time periods ranging from 20 to 50 min with 10 min interval were tested at the optimal amplification temperature. The HBV-LAMP products were analyzed with the MG reagent and LFB. Each of the reaction time was tested independently in triplicate.

### Analytical Specificity of the Hepatitis B Virus–Loop-Mediated Isothermal Amplification-Lateral Flow Biosensor Assay

The HBV nucleic acid standard substance, 13 HBV-positive clinical samples, and 25 non-HBV pathogens (Table 1) were employed for testing the specificity of HBV-LAMP-LFB. The operation was performed as described above. All of the LAMP amplicons were analyzed through the LFB. Each sample was identified at least three times.

### Confirming the Feasibility of the Hepatitis B Virus–Loop-Mediated Isothermal Amplification–Lateral Flow Biosensor Assay Using Clinical Samples

In order to confirm the applicability of HBV-LAMP-LFB assay in clinical setting, 115 serum samples with suspected HBV infection were collected from the Second Affiliated Hospital of Guizhou University of Traditional Chinese Medicine. All of the clinical specimens were evaluated simultaneously using qPCR and HBV-LAMP-LFB. The qPCR diagnosis was carried out using a commercial real-time TaqMan PCR kit (DaAn Gene Co., Ltd. China), and the detection was carried out with the Applied Biosystems™ 7,500 Real-Time PCR system (Life Technologies, Singapore). The concentrations of HBV less than 30 IU will be considered as the negative result according to the manufacturer’s instructions. The HBV-LAMP-LFB operation is as described above.

In the current study, each test was repeated at least three times for repeatability.

## Results

### Overview of the Hepatitis B Virus–Loop-Mediated Isothermal Amplification–Lateral Flow Biosensor Assay System

The principle of HBV-LAMP-LFB assay is illustrated in Figure 1. In brief, the HBV genomic DNA was extracted using nucleic acid–releasing agents within 10 min, and then, preamplification was carried out at a fixed temperature (65°C) for 40 min. We modified the LAMP primers LF and FIP at 5’-end with biotin and FAM, respectively. The HBV-LAMP amplicons were labeled with biotin and FAM; finally, the LAMPs were read out with LFB (Figure 1). For biosensor detection, the dye streptavidin-coated gold nanoparticles (streptavidin-GNPs) were laminated onto the conjugate pad, by adding 1.0 μL of HBV-LAMP products and 100 µL of running buffer (100 mM PBS, pH 7.4 with 1% Tween 20) (Zhu et al., 2021) to the sample pad. The running buffer containing HBV-LAMP products were absorbed, and the detection results were read out visually on the NC membrane (red line) within 2 min. In the positive sample, the biotin/FAM-labeled HBV-LAMP amplicons were captured by anti-FAM at the TL and streptavidin-GNPs were captured by biotin-BSA at the CL. However, in negative results, only the streptavidin-GNPs were captured the biotin-BSA at the CL (Figure 2).

### Confirmation and Verification of Hepatitis B Virus–Loop-Mediated Isothermal Amplification Assay

**FIGURE 4:**
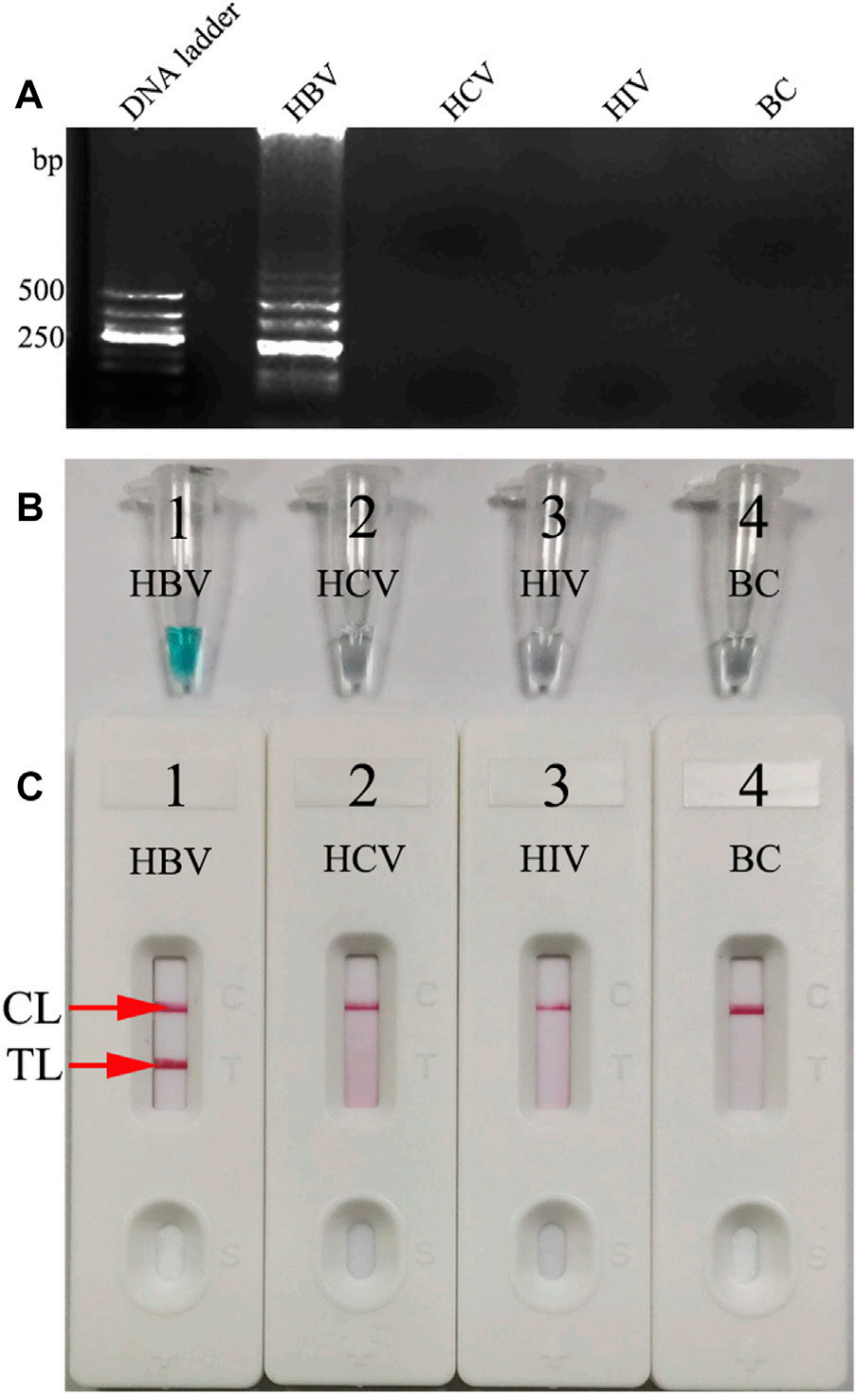
Determination and confirmation of HBV-LAMP products. The HBV-LAMP products were analyzed by 2% agarose gel electrophoresis **(A)** with the MG reagent **(B)** and LFB **(C)**. Lane DNA ladder: 500-bp DNA ladder; the ladder-like bands indicate positive LAMPs **(A)**. The color change from colorlessness to bright green indicates positive nucleic acid amplifications **(B)**. Two visible red lines (CL and TL) appeared for positive results **(C)**. However, there was no band in gel electrophoresis **(A)**, the color remains colorless **(B)**, and only the CL was observed **(C)** in NC and BC.

In order to identify the availability of the set of LAMP primers for the HBV assay, the LAMP reactions were performed in the presence and absence of HBV DNA (Chinese Academy of Metrology) as the template at a constant temperature of 64°C for 1 h. Then, the LAMP products were monitored simultaneously by 2% agarose gel electrophoresis, with a colorimetric indicator (MG) and LFB. The results showed that the positive outcomes appeared with the nucleic acid from HBV, but not from HCV, HIV, and BC (Figures 4A–C). Therefore, these results suggested that the set of HBV-LAMP primers for *S* gene designed here was valid for the development of the HBV-LAMP-LFB assay.

### Optimal Reaction Temperature for Hepatitis B Virus–Loop-Mediated Isothermal Amplification Assay

**FIGURE 5:**
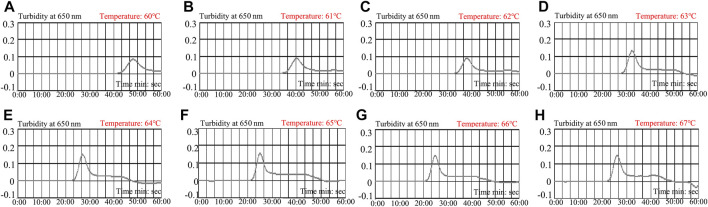
Optimization of amplification temperature for the HBV-LAMP primer set. The LAMP reactions for the detection of HBV were monitored through real-time turbidity (LA-500), and the corresponding curves of DNA concentrations are displayed in the graph. The threshold value > 0.1 was considered as the positive result. 8 kinetic graphs were obtained at different temperatures (60–67°C; 1°C interval) with 100 IU target genomic DNA per reaction. The graphs from e to h show robust amplification. The optimal amplification temperature was 65°C.

To determine the optimal reaction temperature at the preamplification stage, a HBV-LAMP reaction was carried out at a range from 60 to 67°C (with 1°C interval) with 100 IU of HBV nucleic acid as the template per test. The results were monitored by real-time turbidity (LA-500). The kinetic data showed that the robust reactions of HBV-LAMP were obtained at the reaction temperature range from 65 to 67°C (Figure 5). Hence, 65°C was selected as the optimal amplification temperature for the HBV-LAMP-LFB assay.

### Sensitivity of the Hepatitis B Virus–Loop-Mediated Isothermal Amplification–Lateral Flow Biosensor Assay

**FIGURE 6:**
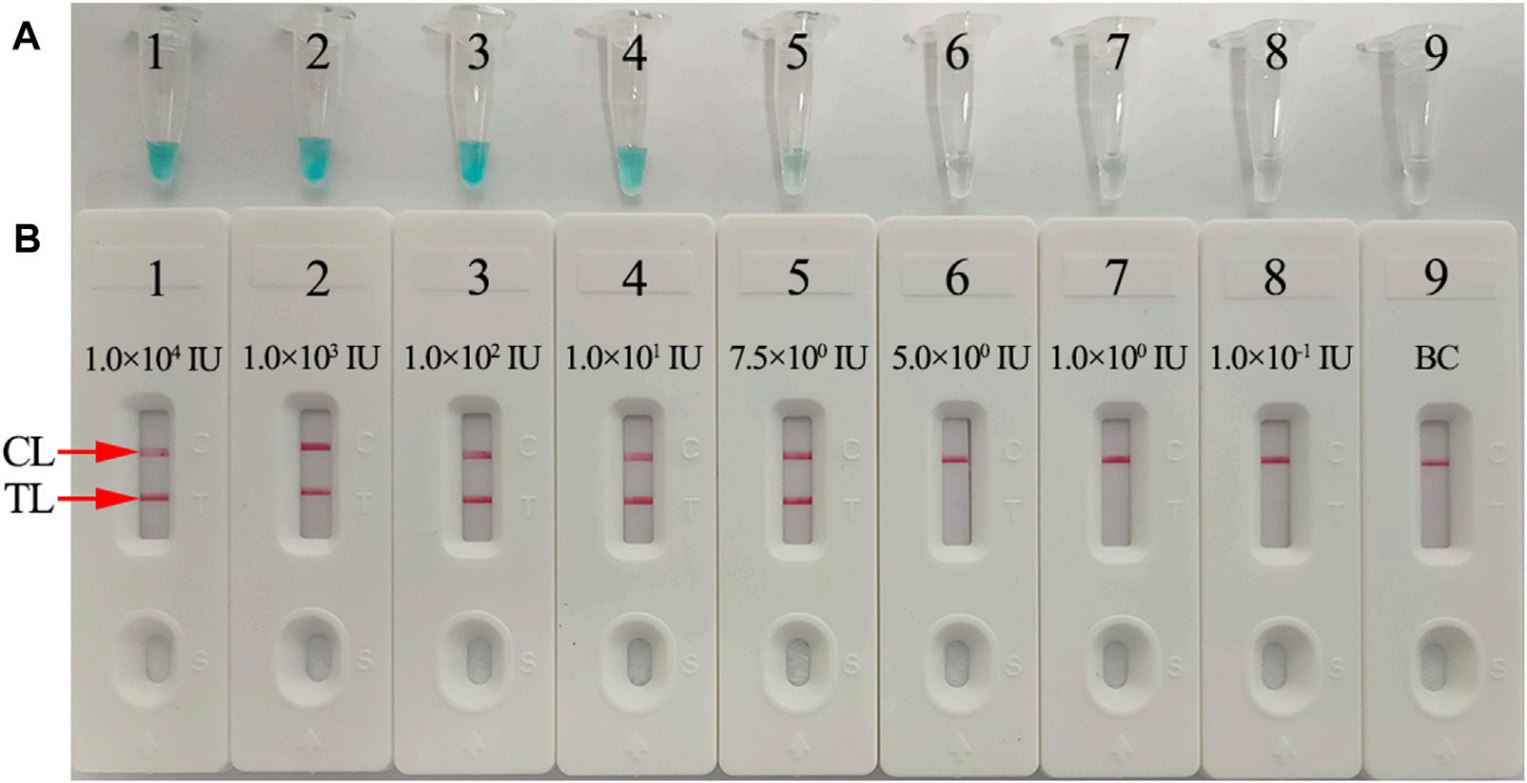
Sensitivity analysis of the HBV-LAMP-LFB assay with serial dilutions of HBV genomic DNA concentrations. Two detection techniques, including colorimetric indicator (MG) **(A)** and LFB **(B)**, were used to analyze HBV-LAMP products. Tubes A1–A9 (Biosensors B1–B9) represent the genomic DNA amounts of 1 × 10^4^ IU, 1 × 10^3^ IU, 1 × 10^2^ IU, 10 IU, 7.5 IU, 5 IU, 1 IU, and 0.1 IU per reaction and BC (DW), respectively. The LoD of the HBV-LAMP-LFB assay for *S* gene detection was 7.5 IU of the genomic template per reaction.

The sensitivity of the HBV-LAMP-LFB assay was verified with serial dilutions of the HBV DNA standard substance (ranging from 1.0 × 10^4^ to 1.0 × 10^−1^ IU) as the template, and the LAMP products were visually read out with the MG reagent and LFB, respectively. As shown in Figures 6A,B, the LoD of the HBV-LAMP assay was 7.5 IU obtained by both detection methods.

### Optimal Amplification Time for the Hepatitis B Virus–Loop-Mediated Isothermal Amplification–Lateral Flow Biosensor Assay

**FIGURE 7:**
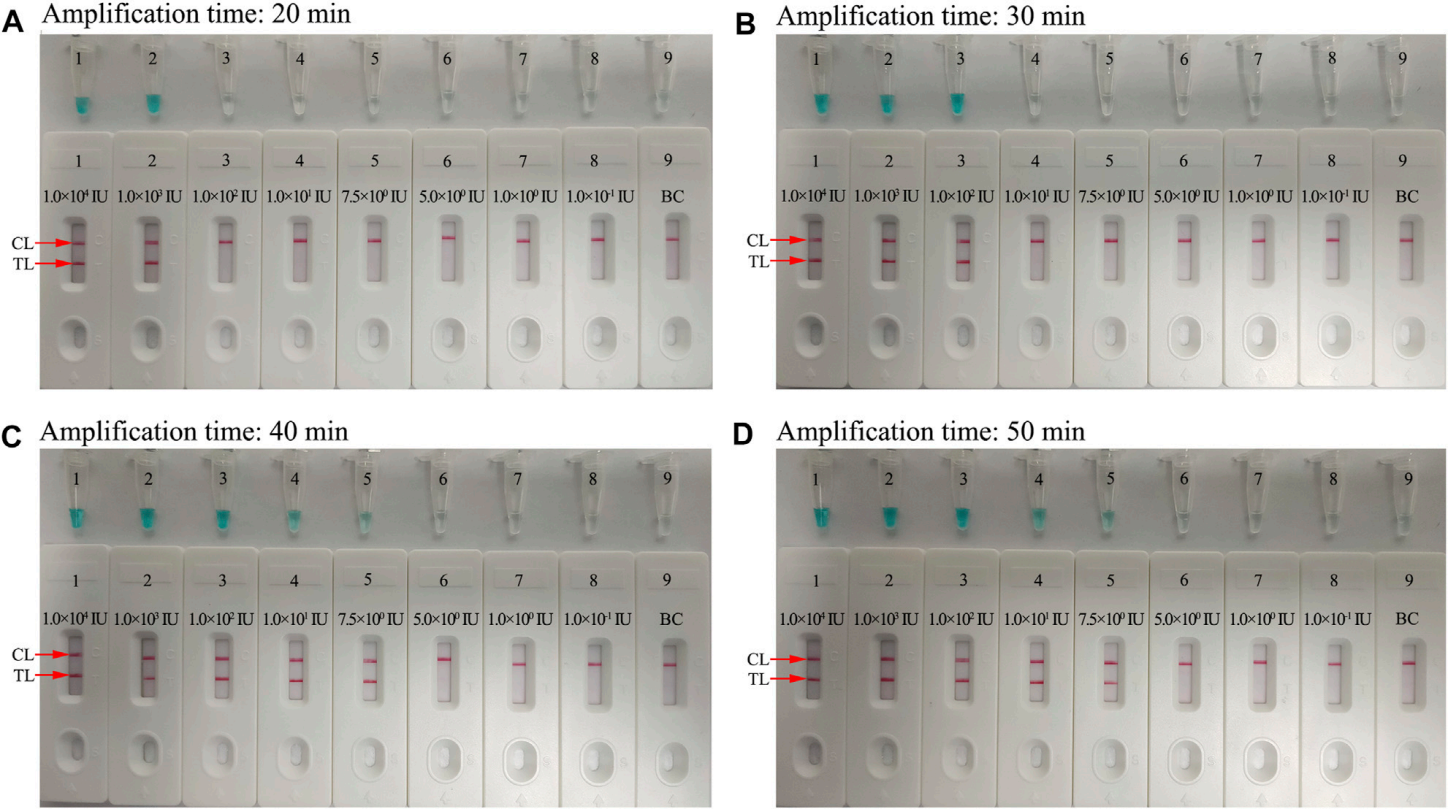
Optimization of the amplification time for HBV-LAMP-LFB detection. Different amplification time intervals, **(A)** 20 min, **(B)** 30 min, **(C)** 40 min, and **(D)** 50 min, were tested at 65°C. Tubes 1–7 (Biosensors 1–7) represent HBV genomic DNA levels of 1 × 10^4^ IU, 1 × 10^3^ IU, 1 × 10^2^ IU, 10 IU, 7.5 IU, 5 IU, 1 IU, and 0.1 IU per reaction and BC (DW), respectively. The best sensitivity was observed when the amplification lasted for 40 min.

To further optimize the reaction time for the HBV-LAMP-LFB assay at the preamplification stage, four reaction time intervals (20, 30, 40, and 50 min) were tested at 65°C, respectively. The LAMP products were identified through MG and LFB methods, respectively. The results confirmed that the limit level of genomic DNA (7.5 IU per reaction) was tested when the reaction lasted 40 and 50 min (Figure 7). Hence, 40 min was recommended as the optimal reaction time for the preamplification stage of the HBV-LAMP assay. Hence, the whole detection procedure, including genomic DNA extraction (∼10 min), preamplification (40 min), and LFB reading (∼2 min), can be accomplished within 60 min.

### Specificity of the Hepatitis B Virus–Loop-Mediated Isothermal Amplification–Lateral Flow Biosensor Assay

**FIGURE 8:**
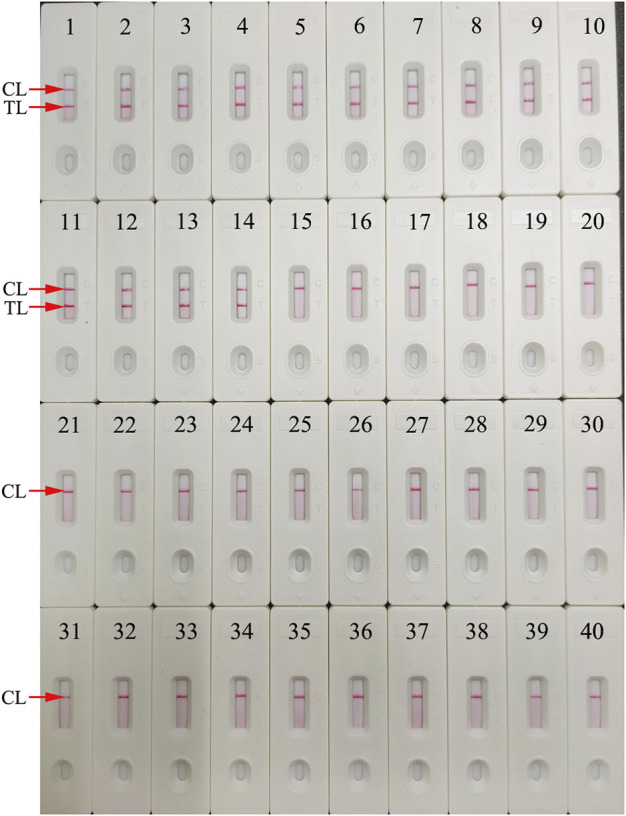
Specificity analysis of the HBV-LAMP-LFB assay using different pathogens. The LAMP reactions were carried out with different genomic RNA/DNA as templates, and each of the amplification products was determined by means of the visual LFB method. Biosensor 1, HBV (standard substance); Biosensors 2–14, HBV (clinical samples); Biosensor 15, HCV (standard substance); Biosensor 16, HIV (standard substance); Biosensor 17, H1N1 (nucleic acid samples); Biosensor 18, influenza B; Biosensor 19, respiratory syncytial virus type A; Biosensor 20, human rhinovirus; Biosensor 21, adenoviruses; Biosensor 22, *Mycobacterium tuberculosis*; Biosensor 23, *Pseudomonas aeruginosa*; Biosensor 24, *Mycoplasma pneumonia*; Biosensor 25, *Streptococcus pneumonia*; Biosensor 26, *Mycoplasma pneumonia* M129/FH; Biosensor 27, *Haemophilus influenzae;* Biosensor 28, *Streptococcus pyogenes*; Biosensor 29, *Staphylococcus aureus*; Biosensor 30, *Acinetobacter baumannii*; Biosensor 31, *Leptospira interrogans*; Biosensor 32, *Shigella flexneri*; Biosensor 33, *Bacillus cereus*; Biosensor 34, *Bordetella parapertussis*; Biosensor 35, *Bordetella pertussis*; Biosensor 36, Enteropathogenic *Escherichia coli*; Biosensor 37, *Candida glabrata*; Biosensor 38, *Cryptococcus neoformans*; Biosensor 39, *Hemophililus parainfluenzae*; Biosensor 40, BC (DW).

For evaluation of HBV-LAMP-LFB assay specificity, the HBV nucleic acid standard substance (Chinese Academy of Metrology), 13 HBV-positive clinical samples, and 25 non-HBV pathogens (Table 1) were used in the current study. The process of HBV-LAMP-LFB assay was as described above. The results showed that only genomic DNA extracted from HBV samples presented positive results and there was no cross-reactivity with other pathogens (Figure 8). Hence, these results indicated that the HBV-LAMP-LFB assay can accurately differentiate an HBV agent from other microbes.

### Evaluation of the Feasibility of the Hepatitis B Virus–Loop-Mediated Isothermal Amplification–Lateral Flow Biosensor Assay With Clinical Samples

To further demonstrate the feasibility of HBV-LAMP-LFB assay as a valuable method for the detection of HBV agents, 115 clinical serum samples were collected from patients and tested simultaneously using the HBV-LAMP-LFB and qPCR assay. Among them, 76 of 115 clinical samples were confirmed as positive results through qPCR (>30 IU), which have also been verified as positive results through the HBV-LAMP-LFB assay. More importantly, 2 of the 39 “negative samples” (approximately 20 IU) were tested as positive outcomes using the HBV-LAMP-LFB assay (Table 3 and Supplementary Table S1). These results suggested that the HBV-LAMP-LFB assay devised here shows more sensitivity in detecting HBV-infected patients, especially for those at the early stage of HBV infection.

**TABLE 3:** Comparison of qPCR and LAMP-LFB methods to identify HBV in clinical samples.

Detection method	Clinical samples (*n* = 115)		
	Positive	Negative	Time consumption
qPCR	76 (>30 IU)	39 (37, <7.5 IU; 2, approximately 20 IU)	Approximately 130 min
LAMP-LFB	78	37	Within 60 min

*Note.* The qPCR diagnosis was carried out using the commercial real-time TaqMan PCR kit (DaAn Gene Co., Ltd., China). The concentrations of HBV less than 30 IU will be considered as negative results according to the manufacturer’s instructions.

## Discussion

HBV infection remains one of the main public health issues worldwide (Nguyen et al., 2020; Coffin et al., 2019). It can lead to a wide spectrum of liver diseases, such as acute hepatitis, chronic hepatitis, cirrhosis, and hepatocellular carcinoma (Nelson et al., 2016; Loomba and Liang, 2017). Besides, HBV, HCV, and HIV co-infections become increasingly frequent owing to shared routes of transmission and resulting in serious public health burden, especially in underprivileged regions of the world (Sarmati and Malagnino, 2019; Leoni et al., 2018). Screening and diagnosis of HBV remain a considerable challenge in many resource-limited settings with cost and equipment limitations (Mallika, 2015). In these regions, screening of HBsAg in blood with ELISA is the main approach for detecting HBV. However, this assay often poses a serious threat of misdiagnosis and transmission of HBV with low sensitivity (Coffin et al., 2019). In recent years, NAATs have become the primary tests for the identification of HBV in clinical application (Zhao et al., 2005; Li et al., 2018). Nevertheless, their use in resource-limited regions is not readily affordable, owing to requiring special facilities and trained experts. Here, the novel HBV-LAMP-LFB assay, integrating the gold nanoparticle-based LFB with preamplification, was devised first for the detection of HBV agents. Achieving this assay just requires exceedingly simple facilities, such as heating block and water bath that can hold a fixed reaction temperature of 65°C for 40 min. In addition, the gold nanoparticle-based LFB provides an easy-to-operate detection platform, which can rapidly and visually read out the HBV-LAMP outcomes. The whole HBV-LAMP-LFB detection process, including genomic DNA extraction (approximately 10 min), preamplification (40 min), and LFB reading (approximately 2 min), can be accomplished within 60 min.

LAMP, as a rapid, sensitive, low equipment–cost assay, was devised first in 2000 by Notomi et al. (2000), and it has been extensively used to detect various pathogens (Dolka et al., 2019; Huang et al., 2017; Kanitkar et al., 2016). The isothermal amplification of the target gene is completed by using a set of four (or six) specific primers spanning six (or eight) distinct regions of the special sequence with *Bst* DNA polymerase at a fixed reaction temperature (between 58 and 69°C) (Notomi et al., 2000). In the current study, three pairs of HBV-LAMP primers were successfully designed and they specifically identified eight regions of the target gene *S* (Figure 3). The specificity of the HBV-LAMP-LFB assay was strongly verified using the nucleic acid extracted from HBV clinical samples and other various pathogens. The positive results only appeared in the positive control and HBV clinical samples and there was no cross-reactivity with other pathogens (Figure 8). Apart from its remarkable specificity, the newly devised HBV-LAMP-LFB assay was able to detect the concentration as low as 7.5 IU (Figure 6), which is sufficient for the diagnosis of HBV agents in clinical application. Moreover, 2 of the 39 clinical “negative samples” (HBV concentrations below 30 IU) were tested as positive outcomes with the HBV-LAMP-LFB. The results indicated that our HBV-LAMP-LFB assay has higher sensitivity than that of qPCR (Table 3 and Supplementary Table S1).

In the current study, the gold nanoparticle-based LFB was successfully designed and used to read out the HBV LAMP reactions. Gold nanoparticles are the most appropriate materials for making a biosensor, owing to their high adsorption, high surface-to-volume ratio, good biocompatible, and easy-to-manipulate features (Aldewachi et al., 2017; Fuentes et al., 2016; Quesada-Gonzá lez and Merkoçi, 2015). The LFBs have been powerfully proven and widely applied for rapid and sensitivity detection of disease, drug abuse, and food safety in resource-limited areas (Huang et al., 2019; Jiang et al., 2021; Huang et al., 2021). Here, the LFB can visually read out HBV-LAMP reactions for labeling with biotin-BSA and anti-FAM probes on the biosensor pads. For positive outcomes, the two crimson red bands (CL and TL) appeared simultaneously on the LFB strip, while only the CL was presented on the LFB, indicating negative results. Although the real-time turbidity and colorimetric indicator could be used for detecting HBV-LAMP amplicons, the former assay requires a high cost for specific facility and the latter method is ambiguous when the concentration of the amplicons was low (Figure 6). The biosensor with high sensitivity, easy-to-operate, and cost-saving (∼€1.7 euros) advantages was considered as the appropriate method to readout the HBV LAMP products. The total cost of each test, including genomic DNA extraction (∼€ 0.9 euros), preamplification (∼€2.9 euros), and LFB reading (∼€1.7 euros), is estimated to be € 5.5 euros, which is cheaper than the qPCR technique. By reason of the foregoing, the HVB-LAMP-LFB assay has significant potential to develop a point-of-care (POC) testing for detecting HBV-suspected patients in clinical setting, especially in resource-impoverished regions of the world.

## Conclusion

In the current study, a rapid, visual, sensitive, simple, and cost-saving HBV-LAMP-LFB assay based on the *S* gene was successfully devised for identifying HBV agents. The results demonstrated that the HBV-LAMP-LFB is a highly specific and sensitive diagnostic tool for the diagnosis of HBV in clinical application. It does not require expensive facilities and reagents, and the whole detection process can be achieved within 60 min. Hence, the HBV-LAMP-LFB assay has remarkable potential to establish a POC testing for the reliable and rapid detection of HBV in clinical setting, especially in resource-limited countries of the world.

## Data Availability

The original contributions presented in the study are included in the article/Supplementary Material; further inquiries can be directed to the corresponding authors.

## Abbreviations

BC, blank control; CL, control line; DW, distilled water; FAM, 6-carboxy-fluorescein; GZCDC, Guizhou Provincial Center for Disease Control and Prevention; HBV, hepatitis B virus; HCV, hepatitis C virus; HIV, human immunodeficiency virus; mer, monomeric unit; MG, malachite green; NC, negative control; NCBI, National Centre for Biotechnology Information; nt, nucleotide; POCT, point-of-care testing; second GZUTCM, Second Affiliated Hospital, Guizhou University of Traditional Chinese Medicine; TL, test line; WHO, World Health Organization.
