# Supplementary material for: Nanoparticle-Based Lateral Flow Biosensors Integrated With Loop-Mediated Isothermal Amplification for the Rapid and Visual Diagnosis of Hepatitis B Virus in Clinical Application
Source: Front Bioeng Biotechnol. 2021 Sep 14;9:731415. doi: 10.3389/fbioe.2021.731415 (PMC8477041; doi:10.3389/fbioe.2021.731415)
Supplement: Supplementary file 1 [file DataSheet1.docx]

**Supplementary Materials**

**Nanoparticle-based lateral flow biosensor integrate with loop-mediated isothermal amplification for rapid and visual diagnose of hepatitis B virus in clinical application**

Xu Chen^1,2Δ*^, Shoshi Wang^2Δ^, Yan Tan^3^, Junfei Huang^4^, Xingui Yang^4^, Shijun Li^4*^

1. The Second Clinical College, Guizhou University of Traditional Chinese Medicine, Guiyang, Guizhou, 550003, People’s Republic of China
2. Central Laboratory of the Second Affiliated Hospital, Guizhou University of Traditional Chinese Medicine, Guiyang, Guizhou, 550003, People’s Republic of China
3. Guizhou Provincial Center for Clinical Laboratory, Guiyang, Guizhou, 550002, People’s Republic of China
4. Laboratory of Bacterial Infectious Disease of Experimental Centre, Guizhou Provincial Centre for Disease Control and Prevention, Guiyang, Guizhou, 550004, People’s Republic of China

^Δ^ Drs. Xu Chen and Shuoshi Wang contributed equally to this article.

*Corresponding author:

Xu Chen, xuchen1220@com

Shijun Li, [zjumedjun@163.com](mailto:zjumedjun@163.com)

**TABLE S1:** Comparison of qPCR, and LAMP-LFB assays for detection of HBV using clinical samples

| **Sample NO.** | **qPCR results（IU）** | **LAMP-LFB results** |
| --- | --- | --- |
| Test 1 | 3.89×10^3^ | + |
| Test 2 | 1.07×10^3^ | + |
| Test 3 | 2.39×10^5^ | + |
| Test 4 | 2.0×10^2^ | + |
| Test 5 | 5.85×10^7^ | + |
| Test 6 | 1.01×10^4^ | + |
| Test 7 | 4.68×10^2^ | + |
| Test 8 | 4.07×10^8^ | + |
| Test 9 | 1.04×10^2^ | + |
| Test 10 | 1.28×10^2^ | + |
| Test 11 | 1.99×10^2^ | + |
| Test 12 | 4.82×10^7^ | + |
| Test 13 | 1.27×10^8^ | + |
| Test 14 | 2.6×10^3^ | + |
| Test 15 | 1.21×10^2^ | + |
| Test 16 | 1.49×10^4^ | + |
| Test 17 | 6.75×10^5^ | + |
| Test 18 | 1.21×10^5^ | + |
| Test 19 | 2.93×10^3^ | + |
| Test 20 | 3.69×10^3^ | + |
| Test 21 | 4.9×10^1^ | + |
| **Test 22** | - **(~22)** | **+** |
| Test 23 | 80 | + |
| Test 24 | 1.09×10^4^ | + |
| Test 25 | 3.1×10^1^ | + |
| Test 26 | 3.45×10^2^ | + |
| Test 27 | 3.10×10^2^ | + |
| Test 28 | 56 | + |
| Test 29 | 4.62×10^3^ | + |
| Test 30 | 1.38×10^8^ | + |
| Test 31 | 7.44×10^3^ | + |
| Test 32 | 42  1.2×10^6^ | + |
| Test 33 | 1.43×10^2^ | + |
| Test 34 | 6.09×10^3^ | + |
| Test 35 | 4.73×10^4^ | + |
| Test 36 | 2.23×10^4^ | + |
| Test 37 | 3.04×10^2^ | + |
| Test 38 | 82 | + |
| Test 39 | 2.57×10^4^ | + |
| **Test 40** | - **(~19)** | **+** |
| Test 41 | 2.05×10^4^ | + |
| Test 42 | 2.92×10^5^ | + |
| Test 43 | 1.82×10^4^ | + |
| Test 44 | 1.33×10^8^ | + |
| Test 45 | 4.94×10^7^ | + |
| Test 46 | 4.37×10^2^ | + |
| Test 47 | 8.94×10^7^ | + |
| Test 48 | 5.60×10^2^ | + |
| Test 49 | 7.06×10^2^ | + |
| Test 50 | 1.36×10^4^ | + |
| Test 51 | 7.71×10^3^ | + |
| Test 52 | 2.14×10^4^ | + |
| Test 53 | 1.1×10^4^ | + |
| Test 54 | 2×10^4^ | + |
| Test 55 | 1.32×10^8^ | + |
| Test 56 | 2.93×10^2^ | + |
| Test 57 | 3.68×10^4^ | + |
| Test 58 | 1.32×10^4^ | + |
| Test 59 | 1.09×10^8^ | + |
| Test 60 | 2.29×10^4^ | + |
| Test 61 | 2.64×10^5^ | + |
| Test 62 | 2.45×10^6^ | + |
| Test 63 | 3.92×10^4^ | + |
| Test 64 | 3.71×10^5^ | + |
| Test 65 | 36 | + |
| Test 66 | 67 | + |
| Test 67 | 1.81×10^6^ | + |
| Test 68 | 5.89×10^3^ | + |
| Test 69 | 53 | + |
| Test 70 | 9.21×10^3^ | + |
| Test 71 | 2.2×10^3^ | + |
| Test 61 | 1.95×10^2^ | + |
| Test 72 | 1.45×10^3^ | + |
| Test 73 | 1.03×10^4^ | + |
| Test 74 | 7.16×10^2^ | + |
| Test 75 | 2.8×10^5^ | + |
| Test 76 | 1.38×10^3^ | + |
| Test 77 | 3.48×10^2^ | + |
| Test 78 | 3.71×10^2^ | + |
| Test 79-115 | — | — |

**Notice:** The qPCR diagnosis was carried out using commercial real-time TaqMan PCR Kit (DaAn Gene Co., Ltd. China). The concentrations of HBV less than 30 IU will be considered as negative results according to the manufacturer’s instructions.

+, Positive; —, Negative
